# Evaluation of Usage of a Fracture Risk Assessment by FRAX Tool in Adults With Type 2 Diabetes Mellitus

**DOI:** 10.7759/cureus.35205

**Published:** 2023-02-20

**Authors:** Sílvia Santos Monteiro, Tiago da Silva Santos, Liliana Fonseca, Jorge Dores

**Affiliations:** 1 Endocrinology, Diabetes and Metabolism, Centro Hospitalar Universitário de Santo António (CHUdSA), Porto, PRT

**Keywords:** osteoporosis, fracture risk, fracture, frax, type 2 diabetes mellitus

## Abstract

Background: Fragility fractures are increasingly recognized as a complication of type 2 diabetes mellitus (T2DM). The FRAX-Port® is a calculation tool that assesses the 10-year risk of either major and hip fracture, integrating several clinical risk factors, including T2DM. We aimed to evaluate the fracture risk in adults with T2DM and determine the rate of patients at high risk for fracture under anti-osteoporotic therapy.

Methods: We developed a cross-sectional study, including a convenience sample of adults with T2DM, followed in our tertiary center between 2019 and 2022. Fracture risk was evaluated according to FRAX-Port®.

Results: One hundred adults were included, 54% male, with a mean age of 68.4±9.2 years. Respecting fracture risk factors, 17% had a previous fragility fracture, 12% had a history of hip fracture in their parents, 9% had active alcohol consumption, and 4% had active smoking. Additionally, 17% presented secondary osteoporosis, being the most frequent cause of systemic corticosteroid exposure (10%). Regarding diabetes-specific risk factors, 94% had a diabetes duration longer than five years; HbA1c greater than 7% in 70%; 42% had diabetic retinopathy, 33% had diabetic chronic kidney disease, 18% had peripheral neuropathy, and 7% had autonomic neuropathy; 83% were on insulin, 2% on canagliflozin and 1% on pioglitazone. According to the FRAX-Port®, the median probability of major fracture was 6.8% (IQR 6.9), and hip fracture was 2.4% (IQR 3.9). Fracture risk was high, intermediate, and low at 41%, 15%, and 44%, respectively. Lastly, 56% of participants should undergo bone densitometry and 45% had a formal recommendation to begin an anti-osteoporotic treatment. However, only 6% were under anti-osteoporotic therapy: bisphosphonates (5%) and denosumab (1%).

Conclusions: More than a third of T2DM patients evaluated had a high fracture risk. We found that FRAX-Port® is an easy-to-apply tool, which helps in the decision to perform densitometry or to institute anti-osteoporotic therapy. Given the increasing prevalence of T2DM and the associated risk of falls, this study highlights the need to recognize the fracture risk in these patients, usually a forgotten complication during the screening of risk factors for adverse events in adults with T2DM.

## Introduction

Osteoporosis is the most prevalent metabolic bone disease worldwide. Its prevalence reached 32 million European individuals in 2019, 5.6% of the total European population, representing a major public health concern. Fragility fractures constitute the principal adverse outcome of osteoporosis and often led to impaired function, chronic pain, and either a major decline in the quality of life or even death [1].

Type 1 diabetes mellitus (T1DM) has been associated with reduced bone mass and increased fracture risk. Additionally, bone fragility is increasingly recognized as a complication of type 2 diabetes mellitus (T2DM) [2]. However, the identification and management of fracture risk in the T2DM population remain challenging since bone mineral density (BMD) in these patients tends to be relatively preserved or even higher than in healthy subjects [2,3]. In T2DM, bone microarchitectural changes decrease bone strength and quality, suppress bone turnover and increase cortical porosity. Moreover, individuals with long-standing diabetes have an increased risk of falls and consequent susceptibility to fractures due to hypoglycemic episodes, decreased visual acuity, and impaired mobility and balance [2]. Considering the progressive aging of the population and the increasing prevalence of diabetes, evidence suggests fragility fractures to represent a major, although underdiagnosed, manifestation of this disease. Strategies to effectively identify and promptly intervene in the risk of an osteoporotic fracture within these individuals are mandatory [1].

These concerns about the clinical utility of BMD in the T2DM population justify the need for alternative tools to accurately predict fracture risk in these patients. The Fracture Risk Assessment Tool (FRAX) is a calculation score that assesses the 10-year probability of hip and major - at the spine, hip, forearm, and humeral - osteoporotic fractures, integrating several clinical risk factors [2]. The Portuguese version of the FRAX tool (FRAX-Port®) was created and fully validated for the Portuguese population and can help in the decision to perform densitometry or to institute anti-osteoporotic therapy [4]. This algorithm applies to individuals aged between 40 and 90 years [4]. Only T1DM is included among the secondary causes of osteoporosis in the FRAX score, whereas T2DM is not. Some strategies have been used to optimize the use of FRAX within individuals with T2DM, such as selecting rheumatoid arthritis as an equivalent variable for T2DM in the FRAX [2].

Evidence addressing the risk of osteoporotic fracture in T2DM individuals is scarce. Therefore, we aimed to evaluate fracture risk in adults with T2DM and determine the rate of patients at high risk of fracture under anti-osteoporotic therapy.

## Materials and methods

We performed a cross-sectional study, including a convenience sample of adults with T2DM, followed in our outpatient Endocrinology and Diabetes Clinic between 2019 and 2022. Only individuals aged between 40 and 90 years were included since FRAX is only applicable within this age range. This work refers to an observational study using data from an anonymized database. All data were anonymously collected and analyzed.

The fracture risk factors that are involved in the FRAX model were documented, including age, gender, height, weight, previous fragility fracture (those occurring at skeletal sites usually associated with osteoporosis such as hip, vertebrae, forearm, and humerus, and not caused by external trauma), parental history of hip fracture, glucocorticoid use, current smoking status, alcohol intake, rheumatoid arthritis and causes of secondary osteoporosis (hyperparathyroidism, long-standing untreated hyperthyroidism, hypogonadism or premature menopause, chronic malnutrition or malabsorption and chronic liver disease). Weight (kg) was measured and height (cm) was self-reported.

The 10-year probability of major osteoporotic fracture and hip fracture was calculated by the Portuguese version of the Fracture Risk Assessment Tool, FRAX-Port® (https://www.shef.ac.uk/FRAX/tool.jsp?lang=pt) [4]. Data about BMD were not available and, therefore, this information was not included in the analysis. The FRAX risk was adjusted to T2DM by selecting rheumatoid arthritis as the equivalent variable for T2DM [2]. Fracture risk was considered high if the probability of a major or hip fracture was above 11% or 3%, respectively; intermediate if the probability of a major or hip fracture was between 7%-11% or 2%-3%, respectively; and low if the probability of a major or hip fracture was below 7% or 2%, respectively [4]. BMD evaluation was warranted when pre-BMD evaluation FRAX-Port® estimates were between 7% and 11% for major osteoporotic fracture and between 2% and 3% for hip fracture [4]. Lastly, it was recommended to start anti-osteoporotic therapy in all subjects that satisfied one or more of the following criteria: a) ≥1 fragility fracture of the hip or ≥1 symptomatic vertebral fragility fracture; b) ≥2 fragility fractures, independently of the site of fracture or the absence of symptoms; and c) estimates of FRAX-Port®, without DXA, ≥11% for major osteoporotic fracture or ≥3% for hip fracture [4].

Categorical and continuous variables were presented as percentages with ratios and mean values ± standard deviation or median values (interquartile range [IQR]), respectively. Distribution normality was tested for continuous quantitative variables using both histogram observation and the Kolmogorov-Smirnov test analysis. All statistical tests were performed using statistical software (SPSS version 25.0 for Windows; IBM Co., Armonk, NY).

## Results

One-hundred patients were included, 54% (n=54) male, with a mean age of 68.4±9.2 years. The median time since diabetes diagnosis was 20 years (IQR 11) and the mean HbA1c was 7.9±1.6%. The mean BMI was 28.4±4.6kg/m^2^. Respecting fracture risk factors, 17% (n=17) had a previous fragility fracture, 12% (n=12) had a history of hip fracture in their parents, 9% (n=9) had active alcohol consumption, and 4% (n=4) active smoking. Additionally, 17% (n=17) presented secondary osteoporosis, especially due to the use of systemic glucocorticosteroids (10%), followed by male hypogonadism (2%) and primary biliary cirrhosis (2%) (Table 1).

**Table 1:** Osteoporotic fracture risk factors ACS = Autonomous Cortisol Secretion Results are presented as percentages with ratios

Fracture risk factors		N=100
Previous fragility fracture		17% (n=17)
History of hip fracture in parents		12% (n=12)
Active alcohol consumption		9% (n=9)
Active smoking		4% (n=4)
Causes of secondary osteoporosis	Systemic corticosteroids	10% (n=10)
	Male hypogonadism	2% (n=2)
	Primary biliary cirrhosis	2% (n=2)
	Primary hyperparathyroidism	1% (n=1)
	Adrenal adenoma with ACS	1% (n=1)
	Menopause <45 years	1% (n=1)
	Aromatase inhibitor	1% (n=1)
Diabetes specific risk factors	Diabetes duration >5 years	94% (n=94)
	HbA1c >7%	70% (n=70)
	Microvascular complications	62% (n=62)
	Diabetic retinopathy	42% (n=42)
	Diabetic kidney disease	33% (n=33)
	Peripheral neuropathy	18% (n=18)
	Autonomic neuropathy	7% (n=7)
	Anti-diabetic therapy	83% (n=83)
	Insulin	83% (n=83)
	Canagliflozin	2% (n=2)
	Pioglitazone	1% (n=1)

Regarding diabetes-specific risk factors, 94% (n=94) had a diabetes duration longer than five years; HbA1c greater than 7% in 70% (n=70); 62% (n=62) had microvascular complications; 83% (n=83) were on insulin, 2% (n=2) on canagliflozin and 1% (n=1) on pioglitazone (Table 1).

According to the FRAX-Port®, the median probability of major fracture was 6.8% (IQR 6.9) and of hip fracture was 2.4% (IQR 3.9). Fracture risk was considered high, intermediate, and low at 41% (n=41), 15% (n=15), and 44% (n=44), respectively. Lastly, 56% (n=56) of participants should undergo bone densitometry and 45% (n=45) had a formal recommendation to begin an anti-osteoporotic treatment. However, only 6% (n=6) were under anti-osteoporotic therapy: bisphosphonates (5%, n=5), namely alendronate (4%, n=4) and ibandronate (1%, n=1), and denosumab (1%, n=1) (Table 2).

**Table 2:** Fracture risk assessment and intervention indication according to FRAX-Port® Results are shown as either median with interquartile range (*)  or percentages with ratios

FRAX-Port® calculation tool		N=100	
Probability of major fracture*		6.8% (6.9)	
Probability of hip fracture*		2.4% (3.9)	
Fracture risk	High	41% (n=41)	
	Intermediate	15% (n=15)	
	Low	44% (n=44)	
Indication for bone densitometry		56% (n=56)	
Indication for anti-osteoporotic treatment		45% (n=45)	
Treatment	Anti-osteoporotic therapy		6% (n=6)
	Bisphosphonates		5% (n=5)
	Denosumab		1% (n=1)
	Calcium supplementation		1% (n=1)
	Vitamin D supplementation		10% (n=10)

## Discussion

Bone fragility is increasingly recognized as a complication of T2DM [2]. According to our study, more than a third of T2DM adults evaluated had a high osteoporotic fracture risk, reinforcing this association. As previously stated, the management of fracture risk in the T2DM population remains challenging since BMD tends to be relatively preserved or even higher than in healthy subjects [2,3]. We found out that FRAX-Port® constitutes an easy and effective alternative tool to predict fracture risk within this population and can guide the decision on performing bone densitometry and starting anti-osteoporotic therapy, which is consistent with previous studies [5-7].

Given that T2DM is associated with higher fracture risk, which is independent of the ordinary clinical risk factors, some have already suggested including T2DM in FRAX. To enhance the ability of FRAX in the prediction of fracture risk in patients with T2DM, it has been suggested the equivalent replacement of rheumatoid arthritis with T2DM in the algorithm, decreasing the T-value of the femoral neck by 0.5 SD or adding 10 years of age [2]. In our study, the FRAX risk was adjusted to T2DM by selecting rheumatoid arthritis as the equivalent variable for T2DM. However, it should be noted that some patients can have both diseases, rheumatoid arthritis and T2DM, which limits the use of this strategy within this subpopulation. We also found that the FRAX tool has other important limitations, namely in those patients with another additional cause of secondary osteoporosis, whose fracture risk calculated by FRAX remains the same.

According to the FRAX-Port®, the median probability of major fracture and hip fracture was similar to other previous studies [5,8]. The mean age of our sample was high, certainly contributing to the higher risk of osteoporotic fracture. However, Valentini et al. found approximately a double FRAX major osteoporotic fracture probability (12.1%) and hip fracture probability (4.7%) in a sample with similar mean age (73 years) [9]. Surprisingly, we found that more than a third (41%) of T2DM adults evaluated had a high osteoporotic fracture risk, despite a reduced number of them under pharmacological therapy with either antiosteoporotic agents or calcium and vitamin D supplementation. A previous study by Hu et al. reported a lower rate of high fracture risk (19.4%) in a cohort of 1047 patients with T2DM [10]. We must not forget that the risk-based thresholds for an intervention that we present are based on cost-effectiveness and are appropriate for cheaper treatment schemes, such as alendronate; expensive treatment strategies may need higher thresholds to justify intervention [2,11].

Additionally, we found a high rate of previous fragility fractures (17%), which is consistent with previous studies that reported a rate of previous fragility fractures between 13.8% and 29.9% [5,12,13]. The mechanisms associated with an increased fracture risk within individuals with T2DM are numerous. Firstly, it is known that estrogen levels are reduced in elderly individuals, which significantly decreases the absorption of calcium within the intestine and compromises 1,25-(OH)2 vitamin D3 production in the kidney, leading to secondary hyperparathyroidism and promoting bone reabsorption. Secondly, the adipokines and inflammatory factors produced in the visceral adipose tissue may also increase bone reabsorption. Lastly, non-enzyme-promoting glycosylation usually results in the deposit of advanced glycation end-products within the organic bone matrix, increasing its fragility, and the accumulation of bone-marrow fat may also increase the risk of fracture [10].

Surprisingly, in our study diabetic specific risk factors were significantly prevalent, although none of them are considered in the FRAX tool. Previous studies have already reported the relationship between poor glycemic control and the risk of fracture, showing that HbA1C levels equal to or above 7% were associated with a higher incidence rate of hip fractures [9,14,15]. Some have stated that a biphasic response may occur within individuals with T2DM during their disease, presenting a decreased risk of fracture close to their diagnosis which only significantly increases after five years. This may be due to a protective effect from both an increase in total fat mass and insulin levels, which leads to an anabolic status that assures normal bone formation in these newly diagnosed T2DM individuals [16]. However, in T2DM, other factors than glycemic control and longer duration of diabetes may affect fracture risk and should be considered, such as diabetic neuropathy, diabetic retinopathy [17], and glucose-lowering agents, such as insulin and thiazolidinediones, which are associated with either a higher risk of hypoglycemia-induced falls and bone loss, respectively, and therefore contribute to an increased risk of fracture [18,19]. Thiazolidinediones interact with peroxisome proliferator-activated receptor (PPAR)γ, favoring adipocyte differentiation over osteoblasts and regulating the expression of genes involved in adipogenesis, glucose homeostasis, and inflammation [2]. Evidence from the CANVAS study on sodium-glucose cotransporter 2 (SGLT2) inhibitors showed a decreased bone density and a higher risk of fractures in patients treated with canagliflozin [20]. Robust evidence of the safety of SGLT2 inhibitors within bone health is warranted, although both empagliflozin and dapagliflozin are currently being favored in T2DM patients with known bone fragility, as the available data on both of them has not raised similar concerns [2]. Furthermore, hypoglycemic episodes increase the risk of falls and consequent fractures [21]. In our study, data about the characteristics of diabetes were available; however, we could not evaluate their effect on fracture risk calculated by the FRAX tool. Ordinary clinical risk factors can be applied to efficiently identify patients with diabetes at increased fracture risk, although risk assessment tools such as FRAX do not fully capture these increased risks, even when BMD is included in the risk score, and thus systematically underestimate the risk of osteoporosis-related fractures in patients with T2DM [2,22,23]. Therefore, the FRAX model should be interpreted with caution and further improved to evaluate the risk of major osteoporotic fractures and hip fractures in theT2DM population.

Our study has several strengths. Given that published studies on this subject are scarce, we consider that our study adds valuable data. Moreover, we had detailed information about diabetes-specific risk factors, which enriched our study. We investigated the absolute risk of fracture in patients with T2DM through the FRAX algorithm, without the evaluation of femoral BMD; this strategy allowed us to decrease the cost associated with auxiliary diagnostic exams, and facilitated our workflow, possibly reflecting a way to the widespread use of this easy and cheap tool in every outpatient setting.

On the other hand, this study has also some limitations. Firstly, its cross-sectional design raises the issue of selection and information bias. The patients’ sample was drawn in an outpatient clinic setting, so it tightly mirrors real-world clinical practice. Secondly, we only included self-reported fractures, which increases the possibility of some recall inaccuracy, especially within the older subjects. In addition, asymptomatic vertebral fractures were not investigated. Moreover, as a Portuguese study, most of the participants were Caucasian from the Mediterranean area; therefore, our results are not generalizable to other populations. Lastly, we did not include a control group, so we were not able to demonstrate that the FRAX score for both major and hip osteoporotic fractures is higher in the T2DM population than in individuals without diabetes.

## Conclusions

More than one-third of the T2DM patients evaluated had a high fracture risk. Given the increasing prevalence of T2DM and the associated risk of falls, this study highlights the need to recognize the fracture risk in these patients, an often-overlooked complication of diabetes. FRAX-Port® constitutes an easy-to-apply tool, which helps in the decision to perform densitometry or to institute anti-osteoporotic therapy in clinical practice. We suggest that a FRAX adjustment for T2DM may be useful within clinical practice, and here recommend applying this fracture risk assessment tool in individuals with T2DM, selecting rheumatoid arthritis as the equivalent variable for T2DM. Further studies are needed on the evaluation of the structural determinants of bone fragility, to increase the accuracy of these algorithms, either by including disease-specific determinants of fracture, or even BMD parameters like trabecular bone score (TBS) that can somehow quantify the quality of the evaluated bone.
